# Why nanodiamond carriers manage to overcome drug resistance in cancer

**DOI:** 10.20517/cdr.2020.52

**Published:** 2020-10-12

**Authors:** Veronika Benson, Abbas Amini

**Affiliations:** ^1^Institute of Microbiology, Czech Academy of Sciences, Prague 14220, Czech Republic.; ^2^Department of Mechanical Engineering, Australian College of Kuwait, Safat 13015, Kuwait.; ^3^Center for Infrastructure Engineering, Western Sydney University, Penrith, NSW 2751, Australia.

**Keywords:** Nanodiamond, drug carrier, drug resistance, cancer therapy, nanoparticles

## Abstract

Nanodiamonds represent an attractive potential carrier for anticancer drugs. The main advantages of nanodiamond particles with respect to medical applications are their high compatibility with non-cancerous cells, feasible surface decoration with therapeutic and cancer-cell targeting molecules, and their relatively low manufacturing cost. Additionally, nanodiamond carriers significantly increase treatment efficacy of the loaded drug, so anticancer drugs execute more effectively at a lower dose. Subsequently, lower drug dose results in less extensive side effects. The carriers decorated with a targeting molecule accumulate primarily in the tumor tissue, and those nanodiamond particles impair efflux of the drug from cancer cells. Therapeutic approaches considering nanodiamond carriers were already tested *in vitro*, as well as *in vivo*. Now, researchers focus particularly on the possible side effects of nanodiamond carriers applied systemically *in vivo*. The behavior of nanodiamond carriers depends heavily on their surface coatings, so each therapeutic complex must be evaluated separately. Generally, it seems that site-specific application of nanodiamond carriers is a rather safe therapeutic approach, but intravenous application needs further study. The benefits of nanodiamond carriers are remarkable and represent a potent approach to overcome the drug resistance of many cancers.

## Introduction

Cancer resistance represents a major cause in cancer treatment failure. During cancer development, cancer cells adapt different strategies to avoid the toxicity of anticancer drugs including fast drug efflux. Increased efflux eliminates the possibility of actual drug action and eventually, it decreases retention of the drug within the tumor. Then, the excluded drugs are quickly cleared from the organism. Application of a higher drug dose is often associated with undesired serious side effects without real therapeutic benefits. Compared to specialized cancer cells, cancer stem cells (CSC) are highly resistant to any therapy due to their nature and quiescent state. Until now, various nanomaterials have been developed with the intention of increasing the therapeutic efficacy of cancer treatment. Nanomaterials were studied *in vitro* and *in vivo*, and some reached clinical trials. Those include poly (lactic-co-glycolic acid) (PLGA) nanoparticles, metallic nanoparticles, carbon nanotubes and nanoparticles, polymer- and lipid-based materials, and many others^[1-4]^. Generally, it seems that the involvement of nanoparticles in designed drug formulae could be indeed beneficial to fight resistant cancer (and CSC) cells. In this review, we focus on carbon nanoparticles, specifically nanodiamonds, and their capacity to improve drug efficacy with respect to treatment-resistant cancer cells.

## Drug nanocarriers

Nanocarriers can significantly improve the efficacy of cancer treatment by overcoming low retention of anti-cancer drugs in tumor tissue and fast drug clearance from circulation. Additionally, nanocarriers also exhibit decreased off-target toxicity in comparison with free drugs. So far, it seems that interactions of nanoparticles with immune cells (excluding the monocyte/macrophage lineage) are rare^[5-6]^. Among other nanomaterial that were developed for biomedical use, carbon material (nanotubes, graphene, fullerenes, carbon dots, films), and particularly nanodiamonds caught scientists’ attention due to their potential use in drug delivery and bioimaging^[5,7-13]^.

## Nanodiamonds

### Nanodiamond synthesis

Nanodiamonds (NDs) represent a heterogeneous family of nanoparticles in terms of size, shape, or surface potential. Those properties arise primarily from different means of preparation, and they significantly affect nanodiamond particle properties and their optimal final use. The main approaches to obtain nanodiamonds for biomedical use are via detonation [detonation ND (DND)] and growth under high-pressure high-temperature (HPHT)^[14]^. While detonation nanodiamonds are mostly of smaller size (1-10 nm), a positive surface charge, and higher sp2 content; the HPHT particles are larger (35-100 nm), negatively or positively charged, and have a lower sp2 content^[15]^. In addition, HPHT nanodiamonds undergo a milling procedure that equips them with sharp edges instead of a spherical shape. Finally, the HPHT particles are large enough to possess luminescent center(s). The luminescent centers are nitrogen-vacancy centers introduced into milled HPHT nanodiamonds by high-energy irradiation^[16]^.

### Nanodiamond properties and benefits

**Figure 1:**
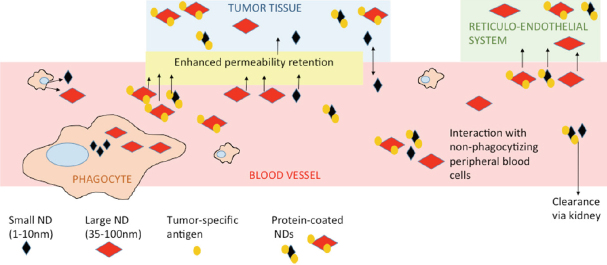
Fate and basic interactions of nanodiamonds after their intravenous administration. The differences between shielded *vs*. unshielded and small *vs*. large nanoparticles are shown. ND: nanodiamond

The size of the nanodiamond influences its circulation time in the blood stream and its accumulation in particular sites. Depending on their final use, we can benefit from small or large particles. Small particles 5 nm in diameter are cleared quickly by the kidneys. Their circulation time in peripheral blood is too short to be accumulated in reticuloendothelial system (RES, particularly liver, kidney, lymph nodes, and spleen) and too short to be effectively accumulated in tumor sites^[17]^. In tumors, the nanodiamonds primarily accumulate via enhanced permeability retention (EPR) or via targeted homing, based on the tumor-specific structure involved in nanodiamond coating^[18,19]^. Larger particles (about 50 nm) cannot be cleared by the kidneys and stay in circulation for long periods. They eventually accumulate in tumor tissue, but they can also accumulate in RES^[17]^. There are reports describing that even though nanodiamonds accumulated at any RES site, they did not cause any substantial harm at the organ or organism levels^[20,21]^. However, there are questions on the accuracy of that statement, such as whether there are any unpublished data contradicting that statement; for example, after using differently prepared or modified carriers that failed in toxicity assays or exhibited any other unfavorable effects. Also, as nano-bio research progresses, we learn more about the potential, pitfalls, and modifications of nanocarriers. A big question in early research was the formation of nanodiamond aggregates, particularly after their intravenous application. Nowadays, different surface coatings or space stabilization have solved undesired aggregation^[10,22]^. The optimal approach seems to be coating with proteins that keep particles relatively dispersed and protect them from excessive protein corona formation. Protein corona forms on the surface of unshielded nanodiamonds after exposure to peripheral blood, or to a lesser extent, after exposure to serum-supplemented culture media^[23]^. The protein structure added as part of the nanodiamond coating will also shield nanodiamond complexes from uptake by peripheral macrophages^[5]^. Conveniently, if we coat nanodiamonds with a tumor-specific antibody, the nanodiamond will effectively accumulate at the tumor site^[5,24]^. Simplified, the fate of nanodiamond carriers after systemic application is shown in Figure 1.

Surprisingly, nanodiamond aggregation was found to be beneficial concerning cancer resistance. Here, controlled agglomeration of nanoparticles overcame the mechanism of tumor drug resistance^[25,26]^.

One of the failure points in drug delivery is the inability of drug carrier to escape from the endosome into the cytoplasm after intake by a cancer cell. The HPHT nanodiamond carriers effectively delivered their cargo into the cell cytoplasm. Chu *et al.*^[27]^ suggested that nanodiamonds were successful in escaping endosomes because they possessed sharp edges, and potentially positive surface charge. After unloading cargo within the endosome, both the sharp edges and the positive charge of the nanodiamonds destabilize the endosomal membrane, enabling the escape of the nanodiamonds and their cargo into the cytoplasm^[10]^. Subsequently, the nanodiamonds usually accumulate in the cytoplasm, close to the nuclear envelope, but rarely enter the nuclei^[8,10,11,28-30]^. There is a recent report regarding the detection of nanodiamonds in nuclei^[31]^; however, it is more likely that the nanodiamonds were embedded within the nuclear membrane as described by Gismondi *et al.*^[32]^. A different situation may occur in the case of coated nanodiamonds. For example, Martín *et al*.^[33]^ proposed that fenton-treated nanodiamonds were able to enter directly into cell nuclei.

The shape of the nanodiamond significantly affects its internalization and accumulation within the cell. We have discussed that sharp edges enable the nanodiamond to escape into the cytoplasm and accumulate there. On the other hand, if the particle edges are less sharp or the nanodiamond is spherical, then the carrier resides in the endosome until it matures to a lysosome, and subsequently the nanodiamond will be expelled from the cell via exocytosis^[27]^.

Different methods of nanodiamond preparation result in particle with positive or negative surface charge. According to their subsequent use, each particle is modified, and total surface charge changes. Positively charged particles can be directly linked with negatively charged drugs or macromolecules. The nanodiamond exhibiting a total negative charge is often coated with a positively charged polymer that enables electrostatic binding of an anionic drug/biomolecule, and the cationic polymer promotes nanocarrier entry into cells^[34]^.

Concerning cell compatibility, one of the key parameters is the amount of sp2 conformation. The level of sp2 contamination depends heavily on the preparation method, and it decreases by optional nanoparticle oxidation. In contrast to sp3, sp2 conformation is less cell compatible. And it has been the probable cause of lower cell-compatibility of detonation nanodiamonds^[15]^. Detonation nanodiamonds contain a high amount of sp2 on their surface due to their preparation method.

Next to specific characteristics resulting from preparation procedure, several properties are common to most nanodiamond carriers. The nanodiamonds are chemically stable, possess rigid structures with octahedral symmetry, and have a large surface area. Regarding sustainable production, their fabrication is relatively low cost and scalable^[28]^.

There are remarkable advantages of nanodiamond particles over other available organic or inorganic carriers. Mainly, nanodiamonds are very flexible regarding surface decoration, and nanodiamonds with luminescent centers are easily traceable. Specifically, surface versatility makes them superior even to synthetic polymeric nanocarriers such as PLGA particles that have already used for drug delivery in clinical application^[4]^. The final, yet important benefit of nanodiamonds is their great compatibility with different live objects. Both naked and coated nanodiamonds were well tolerated by many different cell types including immune cells and specialized tissue cells^[29,30]^. No threatening side effects were found even after long-term persistency of naked nanodiamonds within the body^[21]^. We and others^[5,35]^ have found interactions of coated nanodiamonds with specialized cells, but they did not seem to promote any pathology. It is important to mention though that the interaction of nanoparticles (including nanodiamonds) with specialized cell types are still under extensive research. As the research community witnesses the huge potential of nanocarriers particularly for anticancer therapy, maintaining high scrutiny over these nanocarriers is very important. However, for the safe use of nanocarriers, we need more information regarding particular carrier types and their surface modifications.

Unfortunately, the reported data are rather heterogeneous and represent a significant issue for comparing the behavior of nanodiamond carriers. Nanodiamond characterization parameters and toxicity evaluation methods are not united, and there remains a lack of important information (for example size, method of preparation, and charge) in many reports. Generalized interpretation of such outputs is complicated, because as discussed earlier, size, shape, surface charge, method of preparation, method of coating, and type of coating all affect the final behavior and nano-bio interaction of the carriers.

### Nanodiamonds as drug carriers

Nanodiamonds have found use in many technological approaches and biomedical areas including imaging, microbial resistance management, bone tissue engineering, and root canal fillings^[15,36]^. Overall, their application as drug carriers is the most prevalent. The main reason is that nanodiamonds can be easily functionalized to deliver a wide range of therapeutics and target specific cancer cells. In parallel, concerning acute toxicity, most nanodiamond-based complexes are well tolerated^[9,10,20,29,30]^. The effectiveness of drug adsorption and desorption on/from nano-carriers depends on diamond core purity, surface chemistry, and dispersion quality, as well as on environmental factors - ionic composition, pH, and temperature^[12]^.

As discussed earlier, the nanodiamond surface can be decorated with different functional groups enabling interactions with water molecules or biologically relevant conjugates^[4]^. Nanodiamonds carrying anticancer drugs promote drug accumulation at the tumor site by passive or active mechanisms. The passive mechanism of drug accumulation is based on enhanced permeability and retention effect due to undeveloped tumor vasculature^[37]^. The larger size of carrier-drug is advantageous for the drug accumulation. The nanodiamond carriers possessing a targeting moiety on their surface promote drug accumulation due to active targeting of tumor-specific antigens^[38]^.

Anticancer drugs often linked to the nanodiamond surface are anthracyclines (specifically doxorubicin), daunorubicin, and epirubicin. Anthracyclines are DNA intercalating agents, exhibiting high effectivity in tumor growth suppression; however, they are also extremely toxic. Their major limitation is dose-dependent side effects such as myelosuppression, cardiotoxicity, and the development of acute myeloid leukemia^[39]^. Many reports have shown that the binding of anthracyclines to the nanodiamond carriers significantly reduced the effective dosage, resulting in lesser side effects^[40-44]^. The first reports describing transportation of doxorubicin by nanodiamonds used drug physisorption onto nanoparticles. That approach enabled the easy binding and release of the drug without any chemical modification or active targeting^[45,46]^. Later on, Moore *et al*.^[42]^ and Zhang *et al*.^[47]^ decorated the nanodiamond surface with antibodies recognizing epidermal growth factor receptor (EGFR) on the surface of breast cancer cells. That modification promoted targeted delivery of doxorubicin-nanodiamond complexes into EGFR positive cancer cells.

Since the anthracyclines administered alone triggered serious side effects, the researchers focused also on the premature release of the drug from the nanodiamond core in response to different ambiances^[43]^. Especially important was the complex stability after exposure to sera proteins because early drug release in peripheral blood could trigger the above-mentioned toxicity, similar to the free drug. Wang *et al.*^[43]^ studied epirubicin release from nanodiamond carriers after exposure to sera proteins under physiological pH of blood. They found no epirubicin release to sera up to six hours after application. After cancer cells internalized the complexes, acidic intracellular ambiance, together with intracellular proteins promoted the release of the drug from the nanodiamond core.

Lin *et al*.^[41]^ reported another interesting study that used EGFR-specific monoclonal antibody cetuximab as a targeting molecule. That antibody decorated nanodiamonds delivered paclitaxel into human colon cancer cells *in vivo* (xenograft in nude mice). Paclitaxel is a microtubule inhibitor and its delivery into cancer cells induced mitotic catastrophe and reduced tumor growth^[41]^. Until now, studies covering nanodiamond-mediated drug delivery focused on the efficacy of drug accumulation in tumors or RES, on tumor growth, and sometimes on acute organ toxicity. However, there are only a few studies concerning non-tumoral cells, for example, those residing in the peripheral blood. Those cells are likely to interact to some degree with the applied nanodiamond-drug complexes. Even though the decoration of nanodiamond surfaces with proteins shielded complexes from being engulfed my macrophages^[5]^, some carrier formulations could interact with red blood cells^[35]^ or granulocytes^[5]^. Here we mention exemplary reports describing direct or indirect effects of nanodiamond-drug carriers on different blood elements. Madamsetty *et al*.^[48]^ conducted a complex study of pancreatic ductal adenocarcinoma treatment using nanodiamond cores coated with poly (ethylene glycol) (PEG) and doxorubicin. The authors used a type of pancreatic cancer lacking effective therapy as their model. They showed that doxorubicin carried by a nanodiamond core was more effective than the free drug while exhibiting lower side effects. The complexes triggered reduced tumor growth and accumulated in the tumor site. The authors then considered the interaction of nanocomplexes with blood macrophages. The coated nanodiamond complexes exhibited no toxic effect on the employed macrophages. Unfortunately, the research engaged leukemia cell line PLB-985 instead of the primary culture derived from tissue or peripheral macrophages. Thus, the effect of the nanodiamond-complex on macrophages differs from the *in vivo* situation and may be a concern due to PEG-mediated hypersensitivity^[49]^. Křivohlavá *et al*.^[5]^ used an alternative approach; here, the studied complex contained a nanodiamond core, poly (ethylene imine), interfering RNA, and transferrin as a tumor-specific marker. The authors showed effective accumulation of loaded carriers in tumors after systemic administration followed by the specific knockdown of oncogenic microRNA-135b in tumor cells. The nanodiamond-mediated side effects were evaluated *ex vivo* in aspirates of peripheral blood cells and peritoneal macrophages. After systemic application, plasma samples and tissues were tested as well. In summary, the authors did not confirm any significant toxicity on macrophages, but they pointed out the possible interaction of coated nanodiamonds with granulocytes and splenocytes^[5]^.

It is worth mentioning that not only nanodiamond particles but also nanodiamond scaffolds found application as drug carriers. For example, Suliman *et al*.^[50]^ described the successful delivery and release of bone morphogenetic protein-2 by nanodiamond-based bone transplants.

Remarkably, the nanodiamond-based drug carriers have also been included in the Phenotypic Personalized Medicine-Drug Development (PPM-DD) approach. That platform focuses on de-risked drug development due to the systematic rational design of optimal therapeutic combinations. The PPM-DD platform allows rapid determination of optimal drug combinations and includes both conventional as well as nanocarrier-based approaches^[4]^.

In addition to drug delivery, nanodiamond particles also served in cancer cell imaging. As an example, conjugates of nanodiamond particles with gadolinium (III) were used for magnetic resonance^[51]^. Here, the nanodiamond-gadolinium conjugates increased sensitivity of magnetic resonance in contrast to gadolinium alone. At the same time, using those conjugates enabled lowering the sufficient dosage of gadolinium as well. In addition to improvements in magnetic resonance, nanodiamonds assisted in the detection of stem cells. Wu *et al.*^[52]^ used fluorescent nanodiamonds to label lung stem cells in order to track their engraftment and distribution during the regeneration of lung tissue after injury (murine model).

## Overcoming drug resistance by nanodiamond carriers

### Mechanisms of anti-cancer drug resistance

Treatment resistance to conventional chemotherapy and radiotherapy is a key property of cancer cells that enables their escape and cancer treatment failure^[53]^. Mechanisms of drug resistance differ and encompass the quiescent state^[54]^, fast drug efflux from the cell^[55]^, drug inactivation, mutation of the drug target, enhanced DNA damage repair^[56]^, inhibition of cell death^[57]^, epigenetics, or epithelial-mesenchymal transition^[58]^. The contribution of tumor environment, especially stromal cells, is also important^[59]^.

Drug uptake and drug efflux is regulated mainly by ATP-binding cassette (ABC) protein transporters, including P-glycoprotein (MDR1 or ABCB1). This molecule has been extensively studied due to its fundamental role in multidrug resistance. Overexpression of the ABC transporters correlates with poorer drug response and poorer clinical prognosis^[60]^. The chemoresistance mediated by ABC transporters has been often associated with cancer stem cells, and is likely one of the major mechanisms, why cancer stem cells escape conventional therapy and trigger tumor recurrence^[61]^. Therefore, there is a substantial need to develop new strategies to overcome drug resistance and target cancer stem cells. In some reports, researchers tested targeting of cancer stem cells via stem cells-specific markers such as CD44+, CD90+, CD133+^[62]^, or specific signaling pathways like Notch, Hedgehog, or transforming growth factor-β^[63]^. Recently, the application of nanomaterials has gained additional attention because it offers targeted, controlled, and effective drug delivery and release.

### Nanodiamonds against drug resistant cancer

**Figure 2:**
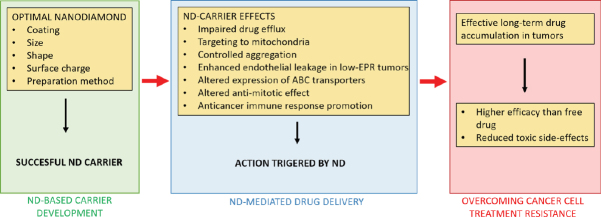
Summary of nanodiamond (ND)-mediated actions that contribute to overcome the treatment resistance of cancer cells

Nanodiamond particles employed as drug carriers possess several properties that help to overcome resistance of cancer cells to conventional therapy. The summarized mechanisms of nanodiamond-triggered cancer cell response are shown in Figure 2.

First, nanodiamonds impair drug efflux from target cells. Cellular transport proteins that pump drugs out of the cells cannot recognize and carry the truncated octahedral structure of the nanodiamond^[40]^. Therefore, the carrier-drug complexes stay inside the cells. Thus, accumulation of the drug inside the cancer cell increases drug efficacy on cancer cells. Simultaneously, drug retention inside cancer cells exposes the nanodiamond-drug complex to the acidic ambiance (low pH) inside the cells that promotes the release of the drug from the carrier. Accumulation and release of the drug inside cancer cells also reduces side effects of drugs on the surrounding tissue^[64]^. For example, Wang *et al*.^[43]^ employed epirubicin linked to 5 nm nanodiamond cores. This complex effectively targeted not only cancer cells, but also cancer stem cells, and thus nanodiamond-based treatment prevented secondary tumor formation in a liver cancer xenograft model. Importantly, the study showed that if linked to nanodiamonds, the epirubicin could be used also in otherwise lethal doses. That means the developed drug formula could be used in patients who could not tolerate the conventional free drug due to its toxic side effects.

Second, the efflux of the anticancer drug by the tumor cells was also bypassed by targeting the loaded nanodiamond carrier all the way to mitochondria via mitochondrial leader sequences (MLS peptide)^[65]^. Chan *et al*.^[65]^ employed fluorescent nanodiamonds loaded with doxorubicin that were simultaneously coated with cancer targeting structures (PEGylated folic acid) and mitochondria targeting MLS peptides. Following endosomal escape, the MLS peptide facilitated transportation of the therapeutic complex directly into mitochondria where doxorubicin induced programmed cell death.

Third, as we have already discussed, nanodiamond size or even the controlled aggregation of nanodiamond-based complexes can be beneficial. The final size of the complex, containing at least a nanodiamond core and drug, usually exceeds the limits for renal clearance, and the loaded nanodiamonds circulate in the peripheral blood for a prolonged time. Increased half-time of nanodiamond-drug circulation maximizes accumulation of the therapeutic complexes within the tumor via an enhanced permeability retention effect, resulting in constant tumor exposure to the drug^[40,66]^. Chang *et al.*^[25]^ were the first to clearly show that self-aggregation of detonation nanodiamond particles significantly contributed to the enhanced therapy efficacy of drug-resistant tumors.

Detonation nanodiamonds were linked with anthracyclines, and their aggregates reached average sizes about 80 nm in diameter. Those aggregates appeared to be critically important for amended tumor therapy. They increased circulatory half-life of the drug-carrier 10-fold, resulting in improved intratumoral drug retention^[25,26]^. Similarly, Toh *et al*.^[67]^ showed enhanced retention of nanodiamond-linked mitoxantrone in chemoresistant breast cancer cells.

Fourth, nanodiamond carriers increase intracellular levels of reactive oxygen species and calcium (Ca^2+^), resulting in enhancement of endothelial leakage. That phenomenon could be used in low-EPR tumors that exhibit reduced EPR-based accumulation of drugs. The nanodiamond carriers encourage permeation of vascular endothelium by the anticancer drug and enable the drug to reach target tumor tissue^[68]^.

Fifth, nanodiamond-anthracyclin complexes altered the expression of the protein transporters responsible for drug efflux. Specifically, nanodiamond cores linked with doxorubicin and PEG decreased the expression of ABCG2, a member of the ABC transporter family^[48]^. The ability of nanodiamond carriers to effectively deliver anthracyclines into cancer cells and overcome their immediate efflux promises a suitable delivery platform to treat cancer stem cells^[43,67]^.

Sixth, nanodiamond carriers altered the anti-mitotic effect of loaded drug citropten. Citropten is a natural compound found in citruses, and free drug application leads to apoptotic cell death of cancer cells. On the other hand, citropten loaded onto nanodiamond carriers interferes with the actin filaments involved in mitosis^[32]^. Therefore, the citropten-nanodiamond complexes inhibited proliferation of rapidly dividing cancer cells but exhibited minimal toxic effect on healthy tissues. Lowering of unfavorable side-toxicity by linking certain drugs to a nanodiamond carrier was discussed above in the case of anthracycline. It could be a useful therapeutic approach in fast growing tumors even though it does not solve the existence of quiescent cancer stem cells.

Seventh, the nanodiamond carriers promoted an anticancer immune response. On this topic, Yuan *et al.*^[44]^ published an interesting study in 2019. They focused on triple negative breast cancer which is characterized generally with poor prognosis and chemoresistance. Nanodiamonds used here were coated with polyglycerol and doxorubicin. Since immunosuppression plays an important role in that cancer, they looked at changes in the tumor environment and adjacent immunological parameters. The doxorubicin coated on nanodiamond carriers did not stimulate the upregulation of P-glycoprotein or interleukin-6, which both act as mediators of doxorubicin resistance. Importantly, the application of nanodiamond-based complexes resulted in the reduced secretion of the granulocyte-colony stimulating factor produced by the tumor and in the reduced production of myeloid-derived suppressor cells (MDSCs). The MDSC are myeloid cells reprogrammed by a tumor to suppress anti-tumor immune responses. This favorable environment led to the activation of macrophages, dendritic cells, and lymphocytes that effectively started an anti-tumor response^[44]^. That study is one of the best reports describing the immunological aspects of nanodiamond application so far. Such complex studies remain rare.

Recent remarkable or important studies have been completed mainly on breast and liver cancers, but there are individual reports describing similar findings in other cancers (e.g., colon, pancreas, lung, and leukemia) too. Tables 1 and 2 summarize exemplary and key studies describing resistance of cancer cells to conventional therapy. Table 1 focuses on studies performed *in vitro* and Table 2 involves studies performed *in vivo* [Tables 1 and 2]. So far, most of the reports suffer from incomplete characterization of the nanomaterial or from suboptimal biological models. It is difficult to perform interdisciplinary research and fulfill ideally both physical chemistry and biology parameters. We have to accept that nanodiamonds represent a heterogeneous material, and each particle modification (size, shape, coating, sp2 amount, *etc*.) will have a specific impact on carrier behavior and final performance. Looking for an ideal formula (concerning desired use) would be easier if there was a united database containing the basic parameters (size, shape, source, preparation, surface charge) of nanodiamond cores as well as the coated constructs, employed biological model, and complex outputs. There is also a bit of concern using different methods for size evaluation. Most reports use hydrodynamic diameter (measured by dynamic light scattering) to characterize the coated nanoparticles; however, some coating combinations, might suffer from weak interaction among nanoparticles (due to macromolecule coating) or formation of larger clusters^[69]^. Then, atomic force microscopy could serve as an alternative method for size evaluation.

**Table 1:** Summary of *in vitro* studies focused on drug - nanodiamond conjugates in order to aim cancer cells that are resistant to conventional treatment

Author	Cancer/model	ND core (size and charge)	Conjugate/size	Conclusion
Chow *et al.*^[40]^ 2011	Liver cancer (Huh7, LTM2)	45 nm (DLS)/17mV	ND-DOX	Decreased DOX efflux/tumor regression
Du *et al.*^[76]^ 2020	Different solid tumors (HeLa, HepG2, MCF-7, CHO)	166 nm (DLS, SEM)/-30 mV	ND-PEG-HYD-FA-DOX/264 nm, -19 mV (DLS, SEM)	Intracellular pH-activated drug release, rapid accumulation in cancer cells
Lin *et al.*^[41]^ 2017	Colon cancer (RKO, HCT116, SW620)	3-5 nm	ND-PTX, ND-PTX-Cet	Induction of mitochondrial cell death
Wang *et al.*^[43]^ 2014	Liver cancer (LT2-MYC)	11 nm/48 mV (DLS)	ND-EPI/89 nm	Prolonged drug retention
Yuan *et al.*^[44]^ 2019	Breast cancer (4T1)	5 nm (DLS)	DOX-PG-ND/84nm	Reverses cancer-induced immunosuppression
Lam *et al.*^[46]^ 2018	Lung cancer (A549, NCI-H460, NCI-H1975)	4 nm/-28 mV (DLS)	ND-GF-PEG, ND-EL-PEG/94 nm, 112 nm	Decreased viability
Zhang *et al.*^[47]^ 2011	Breast cancer (MDA-MB-231)	50 nm/15 mV (DLS)	FND-oligo-PTX-antiEGFR	Specific cancer cell delivery
Madamsetty *et al.*^[48]^ 2019	Pancreatic ductal carcinoma	35 nm	ND-PEG-DOX/76 nm, -10 mV (DLS)	Increased drug efficacy and lower side-effects
Chan *et al.*^[65]^ 2017	DOX-RS breast cancer (MCF-7)	37 nm/-93 mV	FND-MLS-PeFA-DOX /279 nm	Targeting mitochondria, increased DOX uptake
Toh *et al.*^[67]^ 2014	MTX-RS Breast cancer (MDA-MB-231)	23 nm (DLS)/56 mV	ND-MTX	Enhanced drug reflux
Setyawati *et al.*^[68]^ 2016	Primary endothelial cells, MDA-MB-468	5 nm/-24 mV (TEM)	ND and DOX, not combined	Increase of vascular permeability in low-EPR tumors
Man *et al.*^[74]^ 2014	DNR-RS leukemia (K562)	51 nm (DLS)	ND-DNR/93 nm	Increased efficacy
Zhang *et al.*^[75]^ 2014	Gastric cancer (BGC-823)	N/A	(ND-SRF) liposom/128 nm (DLS)	Improved drug bioavailability, decreased tumor growth, suppression of metastasis

Permanent cell lines were used. ND core is characterized with average size and overall surface charge. The method for size evaluation is stated if available. Cet: Cetuximab; FND: fluorescent nanodiamond; DLS: dynamic light scattering, hydrodynamic parameter; DNR: daunorubicin; DOX: doxorubicin; EPI: epirubicin; GF: gefitinib; EL: erlotinib; FA: folate; PeFA: PEGylated folic acid; HYD: hydrazine; PEG: polyethylenglycol; PG: polyglycerol; PTX: paclitaxel; MLS: mitochondrial localizing sequence peptide; MTX: Mitoxantrone; N/A: not available; ND: nanodiamond, unavailable method of ND preparation; RS: resistant; SRF: sorafenib

**Table 2:** Available studies *in vivo* that employed nanodiamonds to overcome cancer cell resistance

Author	Cancer type	ND core (size and charge)	Conjugate/size	Conclusion
Chow *et al.*^[40]^ 2011	Liver cancer (LTM2)	45 nm (DLS)/17 mV	ND-DOX	Decreased DOX efflux/tumor regression
Du *et al.*^[76]^ 2020	Liver cancer (HepG2)	166 nm (DLS, SEM)/-30 mV	ND-PEG-HYD-FA-DOX/264 nm, -19 mV (DLS, SEM)	Specific accumulation in tumor, reduced tumor growth, lower toxicity than free DOX
Lin *et al.*^[41]^ 2017	Colon cancer (RKO)	3-5 nm	ND-PTX, ND-PTX-Cet	Reduced tumor size
Moore *et al.*^[42]^ 2013	Breast cancer (MDA-MB-231)	60 nm (DLS) /near neutral	EGFR - (ND - epirubicin) liposom	Complete tumor regression
Wang *et al.*^[43]^ 2014	Myc-induced liver cancer	11 nm/48 mV (DLS)	ND-EPI/89 nm	Prolonged drug retention
Yuan *et al.*^[44]^ 2019	Breast cancer (4T1)	5 nm (DLS)	DOX-PG-ND/84 nm	Reverses cancer-induced immunosuppression
Zhang *et al.*^[75]^ 2014	Gastric cancer (BGC-823)	N/A	(ND-SRF) liposom/128 nm (DLS)	Improved drug bioavailability, decreased tumor growth, suppression of metastasis

ND core is characterized with average size and overall surface charge. Cet: Cetuximab; FND: fluorescent nanodiamond, HPHT preparation; DLS: dynamic light scattering, hydrodynamic parameter; DOX: doxorubicin; EGFR: epidermal growth factor receptor; EPI: epirubicin; FA: folate; HYD: hydrazine; PEG: polyethylenglycol; PG: polyglycerol; MTX: Mitoxantrone; N/A: not available; ND: nanodiamond, unavailable method of ND preparation; SRF: sorafenib

### Other carbon material against CSC

Other carbon materials such as graphene oxide and carbon nanotubes were also used to overcome tumor chemoresistance, particularly by targeting the cancer stem cells. In the case of graphene oxide, Fiorillo *et al.*^[70]^ suggested that it targeted cancer stem cells based on their phenotype and inhibited several key signal pathways leading to the differentiation of cancer stem cells.

Carbon nanotubes are also a widely studied drug nanocarrier due to their unique properties like membrane penetrability, large drug loading, selective retention in the tumor, generally low toxicity, and photothermic properties. Their limitation for wider use is a thread of unpredictable increase in toxicity due to high impurities content, suboptimal production method, morphology, size, functionalization *etc*.^[71]^.

Carbon nanotubes were considered in two main ways - thermal therapy and drug delivery. Burke *et al*.^[72]^ demonstrated that nanotube-mediated thermal therapy resulted in the impairment of stem cells renewal potential. The nanotubes were targeted against breast cancer stem cells via CD44 marker^[72]^. Yao *et al.*^[73]^ used another approach to eliminate cancer cells; they employed chitosan-coated single wall carbon nanotubes loaded with salinomycin and functionalized with hyaluronic acid.

## Conclusion

Nanodiamonds have several unique properties that make them promising nanomaterial for biomedical applications. These include unique structure, electrostatic properties, a chemically inert core, and a tunable surface. Some nanodiamond properties enable them to challenge cancer cell drug-resistance by overcoming drug efflux, increasing feasible drug dosage while lowering side toxicity, and the effective targeting of cancer stem cells. There are numerous reports of successful *in vitro* and *in vivo* proof of concepts. Reports differ in applied drug-nanodiamond formulation or strategy, and any minor modification in coating or size affects final performance. It is impossible to make a general conclusion pointing out one superior strategy, but the variety of approaches holds great potential. It shows there are many possibilities to combine individual approaches, and tailor therapy to specific cancer type. Clearly, nanodiamond carriers shall remain in focus to accompany and improve conventional chemotherapeutics.

## Declarations

### Copyright

© The Author(s) 2020.
